# New Insights in to the Intrinsic and Acquired Drug Resistance Mechanisms in Mycobacteria

**DOI:** 10.3389/fmicb.2017.00681

**Published:** 2017-04-25

**Authors:** Mohammad J. Nasiri, Mehri Haeili, Mona Ghazi, Hossein Goudarzi, Ali Pormohammad, Abbas A. Imani Fooladi, Mohammad M. Feizabadi

**Affiliations:** ^1^Department of Microbiology, School of Medicine, Shahid Beheshti University of Medical SciencesTehran, Iran; ^2^Department of Biology, Faculty of Natural Sciences, University of TabrizTabriz, Iran; ^3^Applied Microbiology Research Center, Baqiyatallah University of Medical SciencesTehran, Iran; ^4^Department of Microbiology, School of Medicine, Tehran University of Medical SciencesTehran, Iran; ^5^Thoracic Research Center, Imam Khomeini Hospital, Tehran University of Medical SciencesTehran, Iran

**Keywords:** *Mycobacterium*, drug resistance, resistance, tuberculosis, Nontuberculous mycobacteria

## Abstract

Infectious diseases caused by clinically important Mycobacteria continue to be an important public health problem worldwide primarily due to emergence of drug resistance crisis. In recent years, the control of tuberculosis (TB), the disease caused by *Mycobacterium tuberculosis* (MTB), is hampered by the emergence of multidrug resistance (MDR), defined as resistance to at least isoniazid (INH) and rifampicin (RIF), two key drugs in the treatment of the disease. Despite the availability of curative anti-TB therapy, inappropriate and inadequate treatment has allowed MTB to acquire resistance to the most important anti-TB drugs. Likewise, for most mycobacteria other than MTB, the outcome of drug treatment is poor and is likely related to the high levels of antibiotic resistance. Thus, a better knowledge of the underlying mechanisms of drug resistance in mycobacteria could aid not only to select the best therapeutic options but also to develop novel drugs that can overwhelm the existing resistance mechanisms. In this article, we review the distinctive mechanisms of antibiotic resistance in mycobacteria.

## Introduction

Infections due to *Mycobacterium* species are an increasing problem in many countries in the world. This genus contains several important bacteria, which can cause life-threatening infections in human beings. Tuberculosis (TB) is a severe infectious disease caused by *Mycobacterium tuberculosis* (MTB). Non-tuberculosis Mycobacteria (NTM) are also capable of causing serious illnesses in both immunocompetent and immunocompromised individuals. According to the latest report released by the World Health Organization (WHO), there were an estimated 10.4 million new TB cases and 1.4 million deaths from the disease (World Health Organization, 2016). Till now, TB cases caused by drug susceptible strains of MTB, in most instances, can be cured in 6 months using combinations of first-line anti-TB drugs (Horsburgh et al., 2015). According to WHO guidelines, patients with drug susceptible TB should be treated with standard regimen consisting of an initial intensive phase of 2-months of isoniazid (INH), rifampicin (RIF), ethambutol (EMB), and pyrazinamide (PZA) followed by a continuation phase of 4 months of INH and RIF (World Health Organization, 2010).

However, treatment of drug resistant strains, including those of multidrug-resistant (MDR) (defined as resistance to INH and RIF, the two most potent first-line drugs for TB treatment)—and extensively drug resistant-TB (XDR-TB) (defined as *in vitro* drug resistance to INH and RIF plus any fluoroquinolone and at least one of the injectable aminoglycosides), is more challenging, requiring prolonged and expensive chemotherapy (World Health Organization, 2014; Millard et al., 2015). Current WHO guidelines for treatment of MDR-TB recommend that at least four second-line drugs that are likely to be effective, as well as PZA, be administered during the intensive phase of the regimen (World Health Organization, 2011). Therapeutic options for XDR-TB are extremely limited because of resistance to the more potent second line drugs (World Health Organization, 2011).

In the other side, based on reports from many countries in the world, the number of diseases caused by NTMs is also rising (Glassroth, 2008; Nasiri et al., 2015; Wu and Holland, 2015). Yet, all mycobacteria are acid-fast; many species cause lung disease that is often indistinguishable from TB and diagnosis of TB in many parts of the world is still only by sputum smear (Shahraki et al., 2015). Unfortunately, due to the lack of standardized or accepted criteria to define diseases caused by NTM, many NTM cases are frequently misdiagnosed as TB and put on anti-TB medications while treatment of NTM disease is not similar to that of MTB (Brown-Elliott et al., 2012). The newer and more expensive macrolides (i.e., azithromycin and clarithromycin) and quinolones (i.e., ofloxacin and ciprofloxacin) have become the cornerstones of therapy for the most clinically important NTM (Brown-Elliott et al., 2012). Recent studies have also shown the high prevalence of drug resistance in NTM species that threatens adequate control of the disease (Brown-Elliott et al., 2012, 2014; Cândido et al., 2014; Cowman et al., 2015).

With recognizing the scale of the problem, a better understanding of the mechanisms of drug resistance in mycobacterial species will have an important impact on the better application of currently available drugs and will also stimulate the exploration of new targets for development of novel classes of anti-mycobacterial compounds.

## Drug resistance mechanisms in mycobacteria

**Figure 1:**
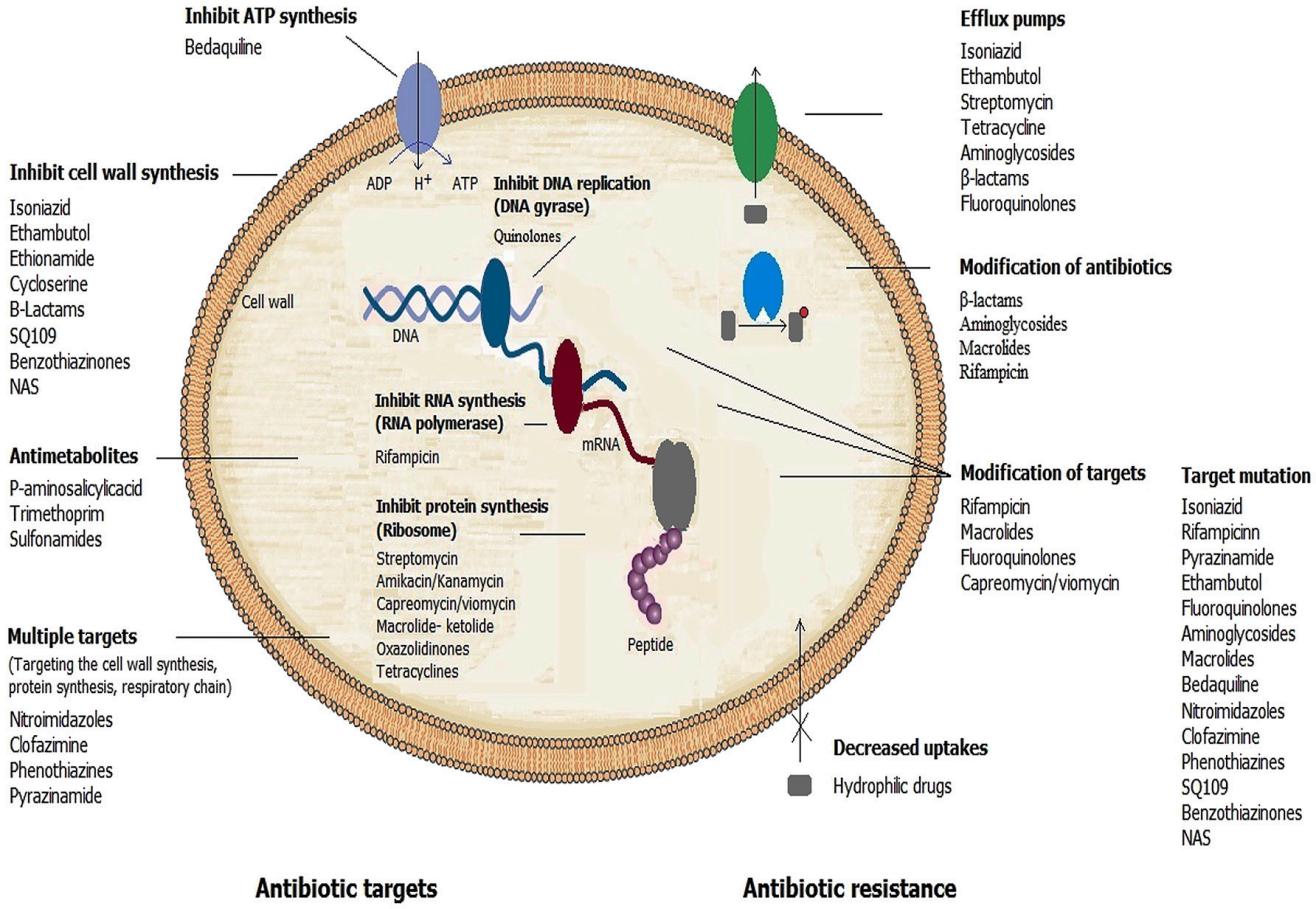
**Mechanisms of action of antimicrobial drugs and ways by which mycobacteria become resistant to them**.

Distinctive mechanisms of antibiotic resistance have been described in mycobacteria. Examples of each of these mechanisms are provided in the following paragraphs (Figure 1).

### Intrinsic resistance

#### Prevention of access to target

##### Cell wall impermeability

The mycobacterial cell envelope consists of three main structural components: (a) a network of peptidoglycan (PG), (b) the arabinogalactan (AG) polysaccharide, and (c) the long-chain mycolic acids (MA) (Alderwick et al., 2015; Daffé, 2015; Jankute et al., 2015). This cell wall-based permeability barrier shields the organism from environmental stress and contributes to resistance of mycobacteria to many antibiotics. Thus, defects in these lipids would damage the function of the cell wall as a barrier and increase the sensitivity to various anti-mycobacterial drugs (Jackson et al., 2013). The enzymes involved in cell wall integrity play an important role in the development of drug resistance in Mycobacterium (Jackson et al., 2013; Xu et al., 2014). Table 1 shows some important enzymes require for integrity of mycobacterial cell wall.

**Table 1:** **Important genes involved in the cell wall integrity of mycobacteria (Philalay et al., 2004; Jackson et al., 2013; Alderwick et al., 2015; Jankute et al., 2015)**.

**Gene**	**Rv number**	**Cellular function**	**Description**	**Final product**
glmU	Rv1018c	PG biosynthesis	UDP-N-acetylglucosamine pyrophosphorylase	UDP-GlcNAc
		LU biosynthesis		
murA	Rv1315	PG biosynthesis	UDP-N-acetylglucosamine enolpyruvyl transferase	UDP-MurNAc
murX	Rv2156c	PG biosynthesis	Phospho-N-acetylmuramoyl-pentapeptide-transferase	Lipid I
ponA1/A2	Rv3682	PG biosynthesis	Lipid II transglycoylase/transpeptidase	Classical (3 → 4)
		Cell wall formation		cross-linked PG
alr	Rv3423c	Alanine metabolism	Alanine racemase	D-alanine
ddl	Rv2981c	Alanine metabolism	d-Alanine–d-alanine ligase	D-alanyl-D-alanine
Ldt	Rv0116c	PG biosynthesis	L,D-transpeptidases	Nonclassical (3 → 3) cross-linked PG
rmlC	Rv3465	AG biosynthesis	dTDP-4-keto-6-deoxyglucose 3,5-epimerase	dTDP-rhamnose
		LU biosynthesis		
accD6	Rv2247	MA biosynthesis	acyl-CoA carboxylases	methylmalonyl-CoA
pks12	Rv2048c	DIM biosynthesis	Polyketide synthase	Dimycocerosyl phthiocerol
ag85	–	MA biosynthesis	mycolyltransferase	trehalose mycolate

*PG, peptidoglycan; AG, arabinogalactan; LU, linker unit; MA, mycolic acid; DIM, Phthiocerol dimycocerosates*.

MurA and MurB are the key biosynthetic enzymes involved in the formation of UDP-MurNAc, an important step in biosynthesis of PG (Alderwick et al., 2015). The naturally occurring broad-spectrum antibiotic fosfomycin is a well-known inhibitor of MurA (Alderwick et al., 2015; Moraes et al., 2015). It specifically inhibits MurA by forming a covalent adduct with a cysteine residue in the active site (Moraes et al., 2015). However, MTB are intrinsically resistant to fosfomycin, as their corresponding cysteine residue is changed into aspartic acid (Alderwick et al., 2015). Conversely, mutation of the wild-type aspartate residue in the MurA active site to a cysteine renders an enzyme sensitive to fosfomycin in MTB (Castañeda-García et al., 2013).

In most bacteria, β-lactam antibiotics inactivate the essential D,D-transpeptidase activity of classical penicillin-binding proteins (PBPs). These enzymes cross-link glycan chains by forming 4 → 3 peptide bonds connecting residues at the fourth and third positions of stem peptides (Gupta R. et al., 2010). However, MTB has been recently found to have a second class of transpeptidases, the L,D-transpeptidases (Ldt) (Gupta R. et al., 2010; Schoonmaker et al., 2014; Basta et al., 2015; Kieser et al., 2015), which are responsible for resistance to β-lactam antibiotics such as amoxicillin and carbapenems (Gupta R. et al., 2010; Alderwick et al., 2015). Till date, five Ldts (*Ldt*_*Mt*1_ to *Ldt*_*Mt*5_) enzymes which form the non-classical 3 → 3 linkages between opposing stem peptides in PG have been identified in MTB (Schoonmaker et al., 2014). MTB strain that lacks both *ldt*_*Mt*1_ and *ldt*_*Mt*2_ displays enhanced susceptibility to amoxicillin and a glycopeptide drug, vancomycin (Schoonmaker et al., 2014).

Moreover, two proteins, PonA1 and PonA2, are bifunctional penicillin-binding proteins that contribute to the biosynthesis and homeostasis of the cell wall components (Jankute et al., 2015). Recent studies indicated that *ponA1* mutant MTB had the same minimum inhibitory concentration (MIC) for b-lactams as wild-type MTB; however, *ponA2* mutant cells were four- to eightfold more susceptible to b-lactams (Shi et al., 2011, 2014; Farhat et al., 2013).

Likewise, any defects in enzymes and proteins that are involved in the cell wall integrity can result in increased susceptibility to multiple drugs. Among the various enzymes in the cell envelope biogenesis, one can find the well-known proteins of the antigen 85 (Ag85) complex (Jankute et al., 2015). These proteins, which show mycolyltransferase activity, are involved in the biogenesis of trehalose dimycolates (TDM), a dominant structure necessary for maintaining cell wall integrity (Jankute et al., 2015). It was shown that inactivation of the Ag85 gene profoundly affects the mycolate content and alters the permeability of the MTB cell envelope (Jackson et al., 1999; Ronning et al., 2000). This change in the permeability of the cell envelope can also result in changes in drug resistance phenotype (Jackson et al., 1999; Nguyen et al., 2005). For instance, individual knockout studies on the Ag85 genes (*fbpA, fbpB*, and *fbpC*) showed that the mutant strains had 40% less mycolate moieties on the cell wall (Torres et al., 2015; Zhang et al., 2017). Nguyen et al. also indicated that the Ag85 mutants display increased sensitivity to both first-line TB drugs as well as other broad-spectrum antibiotics widely used for antibacterial chemotherapy. In their study, they showed that mutant strains of *Mycobacterium smegmatis* were 24-, 85-, 8-, and 12-fold more susceptible to erythromycin, imipenem, RIF, and vancomycin, respectively (Lingaraju et al., 2016). Furthermore, recent studies have shown that the production of TDM by Ag85 is essential for the intrinsic antibiotic resistance of some mycobacteria and support the concept that Ag85-specific inhibitors, alone or in combination with other antibiotics, could provide an effective treatment for TB and other mycobacterial diseases (Nguyen et al., 2005).

Examples of other important proteins include: GlmU, MurX, Alr, Ddl, RmlC, accD6, and Pks12 that were reported to be involved in cell wall integrity of mycobacteria (Table 1; Mills et al., 2004; Philalay et al., 2004; Farhat et al., 2013; Calvanese et al., 2014; Schoonmaker et al., 2014; Alderwick et al., 2015; Jankute et al., 2015). These enzymes were shown to be essential and were attractive targets for the development of new antibacterial agents (Alderwick et al., 2015).

##### Dormancy and latency

Three terms, latency, dormancy and persistence, will be defied here as follow:

Latency refers to a state of asymptomatic infection without apparent disease. Dormancy refers to a non-replicating state with low-to-absent metabolic activity. A subpopulation of dormant cells may be responsible for latency and persistence despite host defense and drug treatment. Persisters, are bacteria that survive antibiotic treatment by remaining dormant even though they are genetically susceptible to these antibiotics (Gomez and McKinney, 2004; Chao and Rubin, 2010).

MTB enters a dormant state during latent infection which is characterized by a shutdown of most of its metabolism causing increased tolerance to antimicrobial agents that are lethal to replicating bacilli a phenomenon referred to as phenotypic drug resistance (Gomez and McKinney, 2004; Gengenbacher and Kaufmann, 2012). In contrast to genotypic drug resistance, this form of drug tolerance is due to reduced metabolic activities or cell division rather than chromosomal resistance mutations (Garton et al., 2008). This is reasoned by the fact that the low metabolic activity in slowly growing or non-replicating dormant MTB is associated with reduced production of antibiotic target proteins or machineries by the bacteria. In fact these bacteria were found to be resensitized upon reentering active growth phase due to resumed production of antibiotic cellular targets (Smith et al., 2012). It's been demonstrated that, *in vitro* induction of dormant state in MTB by oxygen deprivation is associated with shutdown of DNA, RNA and protein synthesis and subsequently increased resistance to anti-TB drugs such as INH and RIF but susceptibility to the anaerobic bactericidal action of metronidazole, which has no effect on aerobic cultures of MTB (Wayne and Hayes, 1996; Hu et al., 1998).

Another system has used nutrient starvation for induced transition of MTB from active growth into a dormant state and revealed decreased respiration and transcription rate, energy metabolism, lipid biosynthesis, cell division as well as concomitant increased drug tolerance to antibiotics targeting functions required for active growth (Betts et al., 2002; Xie et al., 2005). Therefore, the ability of MTB to enter a metabolically inactive dormant phase can be classified as an intrinsic drug tolerance mechanism. Identifying the factors inducing the dormant state and promoting long term survival or persistence of latent MTB might provide novel targets for development of compounds that can treat TB infections by inhibition of these persistence factors. The Ldts enzymes are among the most important factors that plays a vital role in MTB adaptation to stationary phase (Lavollay et al., 2008). As mentioned above, PG of MTB contains a complex network of classical (4 → 3) and non-classical (3 → 3) transpeptide bonds. However, recent studies have shown that upon MTB entry in to the dormancy phase the number of 3 → 3 cross-linkages significantly increases through PG rearrangement by Ldts (Lavollay et al., 2008; Jankute et al., 2015). The rearrangement of PG may have at least two selective advantages. First, Ldts are the only enzymes able to catalyze the formation of new cross-links in the absence of de novo synthesis of precursors (Lavollay et al., 2008). Second, modification of the cross-links may render the peptidoglycan resistant to the hydrolytic activity of endopeptidases (Lavollay et al., 2008). Thus, the development of Ltd inhibitors is a novel and promising approach to obtain drugs for treatment of TB.

Several MTB protein chaperones may also possibly function as a stress responder and consequently contribute to persistence of MTB (Vaubourgeix et al., 2015; Lupoli et al., 2016; Botella et al., 2017). Many of the stresses that MTB encounters, such as reactive oxygen species, changes in pH and mistranslation caused by antibiotics, can cause reversible or irreversible damage to bacterial proteins (Lupoli et al., 2016). In stationary phase, exposure to an aminoglycoside antibiotic like streptomycin, corrupts ribosomal fidelity and induces mistranslation (Dukan et al., 2000; Ballesteros et al., 2001; Vaubourgeix et al., 2015). Recent works suggest that ClpB/DnaK chaperones play unique roles in mycobacteria (Vaubourgeix et al., 2015; Lupoli et al., 2016). These ATP-powered chaperones have been shown to unfold and refold aggregated proteins to their native structures to restore the function of protein (Lupoli et al., 2016). Recent study indicated that ClpB-deficient MTB had a marked recovery defect from stationary phase or antibiotic exposure and survived poorly in mice (Vaubourgeix et al., 2015). In this regard, ClpB and DnaK chaperones in mycobacteria may serve as a novel class of targets for the development of drugs that can sensitize MTB to other drugs (Vaubourgeix et al., 2015).

Another important mechanism which allows MTB to temporarily survive lethal concentration of antibiotics is stochastic variation in the expression of resistance genes. During this phenomenon, MTB persister cells stochastically enter a dormant state, allowing the population to hedge against the appearance of an antibiotic. It is important to know that transient resistance is not caused by genetic changes, rather MTB induces gene expression to generate a resistant phenotype (Wakamoto et al., 2013; El Meouche et al., 2016). For example, stochastic expression of genes affecting antimicrobial action such as catalase-peroxidase *katG*, which activates the prodrug INH accounts for differential antibiotic susceptibility of MTB to INH in a subset of cells (El Meouche et al., 2016; Wakamoto et al., 2013).

##### Porin channels

The permeability of lipid membranes of mycobacteria for hydrophilic solutes is extremely low compared to Gram-negative bacteria. Therefore, hydrophilic agents often utilize channel proteins, such as porins, to cross the outer membrane (Faller et al., 2004; Mailaender et al., 2004; Danilchanka et al., 2008). Porin-like proteins can be detected by channel-forming activities in detergent extracts of MTB and *M. smegmatis* (Heinz and Niederweis, 2000; Raynaud et al., 2002; Niederweis, 2003; Faller et al., 2004; Nguyen and Thompson, 2006; Danilchanka et al., 2008; Song et al., 2008; Niederweis et al., 2010). One of these proteins, MspA has been well characterized and constitutes the major porin of *M. smegmatis* (Niederweis et al., 1999). Deletion of the MspA, drastically increased the resistance of *M. smegmatis* to several agents, indicating that MspA plays an important role in the uptake of hydrophilic antibiotics (Stahl et al., 2001; Stephan et al., 2004). Heterologous expression of the *M. smegmatis* porin gene *mspA* in MTB increasesits susceptibility to hydrophilic antibiotics such as β-lactams as well as anti-TB drugs INH, EMB and streptomycin (Stephan et al., 2004). An understanding of the pathways that enable passage of these drugs through the envelope of MTB is essential to the successful design of new therapeutic agents.

##### Efflux pumps

The intrinsic resistance of mycobacterial species to most antibiotics is generally attributed to the low permeability of the mycobacterial cell wall (Brennan, 2003). Along with cell wall permeability, active efflux systems also provide resistance by expelling the drug molecules that enter the cell. Several mycobacterial drug efflux pumps have been identified and can be grouped into five different structural families: the major facilitator superfamily (MFS) (Siddiqi et al., 2004; Balganesh et al., 2012; Xu et al., 2015), the small multidrug resistance (SMR) family (De Rossi et al., 2002; Garima et al., 2015), the resistance-nodulation-cell division (RND) superfamily (De Rossi et al., 2006; Murakami et al., 2006), the adenosine triphosphate (ATP)-binding cassette (ABC) superfamily (Bhatt et al., 2000; Choudhuri et al., 2002; Pasca et al., 2004; Lubelski et al., 2007; Caleffi-Ferracioli et al., 2016) and the multidrug and toxic compound extrusion (MATE) family (Table 2) (Mishra and Daniels, 2013).

**Table 2:** **Putative MTB efflux pumps associated with reduced susceptibility to antibacterial agents**.

**Efflux pump**		**Gene**	**Resistance to**	**References**
MFS	Tap	*Tap*	STR, TET	Aínsa et al., 1998
	P55	*Rv1410c*	STR,INH, RIF	Silva et al., 2001; da Silva et al., 2011
	Rv1634	*Rv1634*	FQ	De Rossi et al., 2002
	Rv1258c (Tap-like)	*Rv1258c*	STR RIF, OFX, INH	Siddiqi et al., 2004; da Silva et al., 2011
	EfpA	*RV2846* (*EfpA*)	INH, ETH	Doran et al., 1997; Li et al., 2004, 2015
	JefA	*RV2459* (*jefA*)	INH, EMB, STR	Gupta R. et al., 2010; Gupta et al., 2010a,b
	Rv3728	*Rv3728*	INH, EMB, STR	Gupta R. et al., 2010; Gupta et al., 2010a
SMR	MmR	*rv3065* (*mmr*)	INH	De Rossi et al., 1998; Dutta et al., 2010; Rodrigues et al., 2013; Li et al., 2015
RND	MmpL7	*rv2942* (*mmpL7*)	INH	Pasca et al., 2005; Rodrigues et al., 2012
	MmpL5	*rv0677* (*mmpl5*)	Azole compounds	Milano et al., 2009
ABC	Rv0194	*Rv0194*	STR	da Silva et al., 2011
	DrrAB	*drrA* and *drrB*	EMB, FQ, STR, INH, RIF	Choudhuri et al., 2002; Pang et al., 2013; Li et al., 2015
	Rv2686c	*rv2686c*	FQ	Pasca et al., 2004
	Rv2687c	*rv2687c*		
	Rv2688c	*rv2688c*		
	Rv1217	*rv1217*	INH, RIF	Balganesh et al., 2010; Wang et al., 2013
	Rv1218	*rv1218*		
	Rv1456c	*rv1456c*	EMB, STR, INH, RIF	Hao et al., 2011
	Rv1457c	*rv1457c*		
	Rv1458c	*rv1458c*		

*MFS, major facilitator superfamily; SMR, small multidrug resistance; RND, resistance-nodulation-cell division; ABC, adenosine triphosphate-binding cassette superfamily; STR, streptomycin; RIF, rifampicin; OFX, ofloxacin; INH, isoniazid; FQ, fluoroquinolones; ETH, ethionamide; EMB, ethambutol, TET, tetracycline*.

These efflux pumps actively transport many antibiotics out of the cell and are major contributors to the intrinsic resistance of mycobacteria to many drugs. Some efflux pumps have narrow substrate specificity (such as TetV pumps), but many transport a wide range of structurally dissimilar substrates and are known as MDR efflux pumps. The MFS family of MDR efflux pumps is found in many species of mycobacteria and includes several members that are relevant to antibiotic resistance (Yamchi et al., 2015). When overexpressed, MFS pumps confer clinically relevant levels of MDR and export an extremely wide range of substrates.

For example, the increased transcription of jefA, a well-studied example of MFS pumps in MTB, leads to increased resistance to INH, EMB and streptomycin (Gupta et al., 2010b). Likewise, the best-characterized efflux pumps in NTM include the tap in *M. fortuitum* conferring resistance to tetracycline and aminoglycoside or lfrA efflux pumps in *M. smegmatis* that mediate resistance to fluoroquinolones, RIF and INH (Liu et al., 1996; Takiff et al., 1996; Aínsa et al., 1998; Li et al., 2004; Ramón-García et al., 2006; Bowman and Ghosh, 2014). In clinical strains of MTB, increased expression of efflux pumps occurs mainly as a result of induction in response to antibiotic stress (Jiang et al., 2008; Gupta et al., 2010a; Li et al., 2015). For instance, studies that investigated the relevance of active efflux in the drug resistance of clinical strains of MTB indicated that *jefA, drrA, drrB, efpA, mmr*, and RV1217-Rv1218 efflux pumps were overexpressed under INH and RIF stress (Wang et al., 2013; Blair et al., 2015; Li et al., 2015). Furthermore, several studies indicated that some RIF or INH-resistant strains of MTB did not have sequence alterations in the core region of the drug target encoding genes (e.g., *rpoB, katG, inhA*; Yamchi et al., 2015; Manson et al., 2017). Consequently, efflux pumps might play an important role in RIF- and INH- resistance in MTB, especially in those strains having no mutation in genes associated with INH and RIF resistance. Similarly, elevated level of expression in efflux pump genes are also demonstrated to be involved in drug resistance development in NTM species (Rodrigues et al., 2009; van Ingen et al., 2012). Understanding the mechanisms controlling the overexpression is important as it is a common mechanism of resistance in clinically important mycobacteria, and also provides promising target for designing novel class of anti-mycobacterial therapeutics that can treat mycobacterial infections by efflux-pump prevention.

Since efflux pumps play a major role in the development of drug resistance in MTB, many studies have focused on identifying potential efflux pump inhibitors. For example, efflux pump inhibitors reserpine, verapamil, 2,4-dinitrophenol (DNP) and pyrrole derivatives have been shown to increase the susceptibility to norfloxacin, RIF and INH by inhibiting efflux pumps (i.e., Rv1258c and MmpL3), in drug-susceptible mycobacteria (Sharma et al., 2010; World Health Organization, 2011; La Rosa et al., 2012; Machado et al., 2012; Willers et al., 2017). Another efflux pump inhibitor is a mammalian efflux pump inhibitor, timcodar (Nasiri et al., 2016). An adjuvant effect was demonstrated in combination with RIF, bedaquiline and clofazimine when MTB was cultured in host macrophages (Nasiri et al., 2016). Recent studies also demonstrate the potential role of efflux pump inhibitors in treatment regimens to improve and shorten anti-TB treatment (World Health Organization, 2010, 2011).

#### Modification of targets

Intrinsic resistance to several important antibiotics in mycobacteria can also be achieved by modification of target sites. For example, erythromycin resistance methylase (*erm*) gene in MTB encodes an enzyme that methylates a specific site in the 23S rRNA and alters the drug-binding site, thus preventing the binding of macrolides (Buriánková et al., 2004). Subinhibitory levels of clarithromycin have been shown to cause induction of *erm* expression and a 4- to 8-fold increase in MIC values (Andini and Nash, 2006).

These methylases have also been characterized in the clinically important RGM *Mycobacterium abscessus*, as well as in several clinically less relevant NTM species (Nash et al., 2005, 2009; van Ingen et al., 2012; Maurer et al., 2015). Expression of *erm* in *M. abscessus* conferred resistance to clarithromycin, ethromycin and ketolides (Nash et al., 2009; Choi et al., 2012; Stout and Floto, 2012). Thus, macrolide resistance induced by the *erm* gene expression may explain the inefficiency of macrolide based therapy against *M. abscessus* (Nash et al., 2009; Choi et al., 2012; Stout and Floto, 2012).

Another identified example is *mfpA* gene, which confers resistance to quinolones in MTB (Hegde et al., 2005; Tao et al., 2013; Mayer and Takiff, 2014). The *mfpA* gene encodes pentapeptide repeat proteins, which bind to and protect DNA gyrase from the lethal action of quinolones (Hegde et al., 2005). Three-dimensional structure analysis of MfpA showed that its structure displays size, shape, and electrostatic similarity to B-form DNA, and MfpA has been suggested to interact with DNA gyrase via DNA mimicry (Hegde et al., 2005). By binding to gyrase in DNA's place, MfpA apparently divests fluoroquinolones of their target (Ferber, 2005).

Loss of methylation can also confer resistance to certain antibiotics. A well-known example is the resistance to capreomycin and viomycin that occurs when the methyltransferase *tlyA* is deactivated (Maus et al., 2005b). This gene codes an rRNA methyltransferase and that loss of methyltransferase activity yields an unmethylated ribosome that is resistant to these drugs (Maus et al., 2005b). Furthermore, inactivation of GidB, which methylates 16S rRNA, confers low-level streptomycin resistance (Verma et al., 2014).

An intrinsic mechanism by which mycobacteria can increase their tolerance to RIF is the RNA polymerase binding protein A (RbpA), which has been characterized in MTB and *M. smegmatis*. This protein binds to the RNA polymerase, where it hampers binding of RIF (Dey et al., 2010).

#### Modification of antibiotics

As well as preventing antibiotics from entering the cell or altering their targets, mycobacteria can also degrade or modify antibiotics.

##### Enzymatic degradation of antibiotics

Mycobacteria can encode several specific enzymes that have evolved to target and cleave antibiotics of different classes, including β-lactams, aminoglycosides and macrolides (Wang et al., 2006; Da Silva and Palomino, 2011; Wivagg et al., 2014). The β-lactam antibiotics are among the most important classes of antibacterial compounds and act by inhibiting the bacterial transpeptidases that are responsible for the final step of peptidoglycan cross-linking (Wright, 2005). In mycobacteria, β-lactam resistance is primarily due to the production of an Ambler class-A β-lactamase encoded by the *blaC* gene (Hugonnet and Blanchard, 2007). However, other factors such as low cell envelope permeability and presence of low penicillin-binding protein binding affinity for beta-lactams were also believed to contribute to the ineffectiveness of beta-lactams in mycobacteria (Wang et al., 2006; Jankute et al., 2015). Class A enzymes in MTB include the beta-lactamase encoded by the chromosomal *blaC* gene, which shares extensive similarity with many eubacterial b-lactamases (Wang et al., 2006; Nampoothiri et al., 2008). These enzymes are large proteins with much greater penicillinase activity than cephalosporinase activity (Wang et al., 2006). Like other Class A β-lactamases, BlaC hydrolysis the β- lactam ring via nucleophilic attack by an active site serine residue to inactive the drug (Tremblay et al., 2010). In addition, MTB BlaC displays relatively broad hydrolysis spectrum to all β-lactam classes, including the members of the carbapenem antibiotics, which are generally resistant to β-lactamases of other pathogenic bacteria (Hugonnet and Blanchard, 2007; Tremblay et al., 2010). Moreover, β-lactamase inhibitors such as clavulanic acid are less effective against BlaC compared to other class A enzymes (Hugonnet and Blanchard, 2007). Although mycobacterial beta-lactamases can hydrolysis carbapenems, reactions proceed slowly, and one member of the carbapenem family, meropenem, has been reported to be active *in vitro* in association with clavulanic acid against drug resistant strains (Hugonnet et al., 2009; Mainardi et al., 2011). Meropenem may also have potential applications for the treatment of susceptible TB, because meropenem–clavulanic acid is active against nonreplicating forms of bacilli, which are difficult to eradicate even with INH and RIF (Mainardi et al., 2011).

Ambler class A β-lactamases have also been described in *M. fortuitum* and *M. smegmatis* (Nguyen and Thompson, 2006; Soroka et al., 2014).

##### Enzymatic modification of antibiotics

The addition of chemical groups to specific sites on the antibiotic by mycobacterial modifying enzymes causes antibiotic resistance by preventing the antibiotic from binding to its target protein.

Among mycobacterial species, aminoglycoside resistance is sometimes caused by modifying enzymes that are coded by genes on the chromosome (Zaunbrecher et al., 2009; Maurer et al., 2015). Resistance to aminoglycoside antibiotics is also conferred by target mutation, reduced uptake and/or increased efflux (Aínsa et al., 1998; Da Silva and Palomino, 2011). Till now, two main classes of aminoglycoside-modifying enzymes namely, acetyltransferase and phosphotransferase, have been demonstrated in several mycobacterial species (Zaunbrecher et al., 2009; Kim et al., 2011; Ahn, 2013). The best biochemically characterized aminoglycoside-modifying enzyme in mycobacteria is aminoglycoside N-acetyltransferase (AAC 2′) which is capable of acetylating all know aminoglycosides bearing 2′ amino group including neomycin, kanamycin, gentamycin, tobramycin and ribostamycin (Vetting et al., 2002). Distinct N-acetyltransferases have been identified in the genomes of the MTB, *M. kansasii*, the rapid growers *M. fortuitum, M. smegmatis*, and *M. abscessus* (Ho et al., 2000; Ripoll et al., 2009; Ramirez and Tolmasky, 2010; van Ingen et al., 2012).

Another discovery is the presence of a RIF-resistance gene in some mycobacterial species (Baysarowich et al., 2008). In the opportunistic pathogen *M. smegmatis*, RIF is an ineffective drug because of the presence of a chromosomally encoded RIF ADP-ribosyltransferase (Baysarowich et al., 2008). This specific enzyme, transfers the ADP-ribose unit to a hydroxyl residue at position 23 of RIF, rendering the bacterium resistant to RIFs (Baysarowich et al., 2008).

#### Activation of a transcriptional regulator

Intrinsic resistance to any antimicrobial drugs may also be determined by an interactive network, including effector proteins, regulatory proteins, and inducers (Morris et al., 2005; Burian et al., 2012). WhiB7, is a transcriptional regulator that contributes to intrinsic antibiotic resistance in mycobacteria by activating its own expression and many drug resistance genes (Burian et al., 2012). *whiB7* transcription is auto-regulated and its expression can be induced by exposure to sub-inhibitory concentrations of antibiotics as well as a variety of stress conditions such as heat shock, iron starvation, and entry in to stationary phase (Geiman et al., 2006; Burian et al., 2012). Importantly, WhiB7 controls expression of *eis* gene that plays an important role in mycobacterial survival within macrophages (Wei et al., 2000).

A recent condition-specific model analysis also suggests that knocking out and overexpressing of several transcription factors would cause different phenotypes in MTB. For example, this model predicted overexpression of the transcription factor *whiB4* in the presence of ethionamide and INH (Ma et al., 2015).

In addition to WhiB7, MTB also encodes several sigma factors, including SigF that is antibiotic-inducible and plays a part in intrinsic MDR phenotypes (Sharma et al., 2010; La Rosa et al., 2012; Machado et al., 2012).

Other control genes such as *dosR, mbtB* and *hspX* in MTB have also been reported to be implicated in various processes ranging from dormancy to drug tolerance/persistence (Timm et al., 2003; Voskuil et al., 2003; Boshoff et al., 2004; Nandakumar et al., 2014; Sharma and Tyagi, 2016). DosR (dormancy transcriptional regulator) is a well-characterized two component system in MTB which is induced in response to hypoxia and multiple stresses (Kendall et al., 2004; Gautam et al., 2014; Mehra et al., 2015; Sharma and Tyagi, 2016). DosR is believed to be one of the key regulators that mediate MTB survival within granulomatous lesions found in TB (Converse et al., 2009). Recent study indicated that treatment of wild-type MTB with INH resulted in increased induction of *dosR* gene (Nandakumar et al., 2014). These antibiotic-induced responses then may contribute functionally to endogenous antibiotic tolerance in MTB (Nandakumar et al., 2014).

### Acquired resistance

The anti-mycobacterial agents specifically bind to their targets with high affinity, thus preventing the normal activity of the targets. Changes to the target structure that prevent efficient antibiotic binding can confer resistance. Unlike the situation in other bacteria where acquired drug resistance is generally mediated through horizontal transfer by mobile genetic elements, in mycobacterial species, it is caused mainly by spontaneous mutations in chromosomal genes encoding targets. Table 3 provides a summary of drug targets and known or possible mechanisms of resistance in mycobacteria.

**Table 3:** **Anti-mycobacterial drugs and mechanisms of drug resistance**.

**Agent**	**Mode of action**	**Target**	**Proven utility for (MIC μg/ml)**	**Gene**	**Gene function**	**Most prevalent mutation**	**References**
Isoniazid	Inhibition of mycolic acid synthesis	Mycolic acids	*M. tuberculosis (0.02–0.1 in 7H10)*	*katG*	Catalase-peroxidase	Ser-315-Thr	OFFICIAL, 1990; National Committee for Clinical Laboratory Standards (NCCLS), 2000; Somoskovi et al., 2001; Ramaswamy et al., 2003; Zhang et al., 2005; Guo et al., 2006
				*inhA*	Enoyl ACP reductase	C-15-T SNP	
				*Ndh*	NADH dehydrogenase II	Arg-13-Cys, Val-18-Ala	
				*ahpC*	Alkyl hidroperoxidase	C-39-T, G-9-A, SNPs	
				*kasA*	b-ketoacyl-ACP synthase	Gly-269-Ser	
Rifampicin	Inhibition of RNA polymerase	RNA polymerase	*M. tuberculosis (1 in 7H10) M. kansasii (2) M. marinum (1)*	*rpoB*	B-subunit of RNA polymerase	Ser-450-Leu	OFFICIAL, 1990; Heep et al., 2001; Somoskovi et al., 2001; Philley and Griffith, 2015
Pyrazinamide	Inhibition of energy production and trans-translation	Fatty acid synthase-I, ribosomal protein S1	*M. tuberculosis (16–50 in LJ)*	*pncA*	Pyrazinamidase	Asp-12-Ala/Asn,Leu-85-Pro	Somoskovi et al., 2001; Shi et al., 2011; Feuerriegel et al., 2013; Zhang et al., 2013, 2014
				*rpsA*	S1 ribosomal protein	Deletion Ala438, Thr-5-Ala	
				*panD*	Aspartate decarboxylase	Ala-128-Ser, Val-138-Aal	
Ethambutol	Inhibition of arabinogalactan synthesis	Arabinosyl transferases	*M. tuberculosis (5 in 7H10) M. kansasii (5) M. marinum (5)*	*embCAB*	Arabinosyl transferases	Met-306-Val/Ile/Leu	OFFICIAL, 1990; Brown-Elliott et al., 2012; Palomino and Martin, 2014; Philley and Griffith, 2015
Streptomycin	Inhibition of protein synthesis	30S ribosomal subunit	*M. tuberculosis (2–10 in 7H10)*	*rpsL rrs gidB*	S12 ribosomal protein 16S rRNA 16S rRNAmethyltransferase	Lis-43-Arg A-1401-G SNP Leu-16-Arg	OFFICIAL, 1990; Finken et al., 1993; Da Silva and Palomino, 2011; Verma et al., 2014; Philley and Griffith, 2015
Amikacin/Kanamycin	Inhibition of protein synthesis	30S ribosomal subunit	*M. tuberculosis (5 in 7H10)*	*Rrs*	16S rRNA	A-1401-G SNP	OFFICIAL, 1990; Zaunbrecher et al., 2009; Da Silva and Palomino, 2011; Georghiou et al., 2012; Kasperbauer and De Groote, 2015; Philley and Griffith, 2015
			*M. kansasii (32)*	*Eis*	Aminoglycoside acetyltransferase	G-37-T, G-10-A, G-14-T SNPs	
						*M. marinum (32)*	
						RGM (64)	
Capreomycin/viomycin	Inhibition of protein synthesis	30S and 50S ribosome subunits	*M. tuberculosis (10 in 7H10)*	*rrs*	16S rRNA	A-1401-G SNP	Johansen et al., 2006; Da Silva and Palomino, 2011; Georghiou et al., 2012
				*tylA*	rRNA methyltransferase	G-223-T SNP	
				*Eis*	Aminoglycoside acetyltransferase	G-37-T, C-12-T SNPs	
Ethionamide	Inhibition of mycolic acid synthesis	Mycolic acids	*M. tuberculosis (5 in 7H10)*	*ethA*	Monooxygenase	Leu-397-Arg, Leu-328-Met	Morlock et al., 2003; Boonaiam et al., 2010; Brossier et al., 2011
				*inhA*	Enoyl-ACP reductase	Ile-21-Thr/Val, Ser-94-Ala	
				*Ndh*	NADH dehydrogenase	Arg-13-Cys, Val-18-Ala	
				*mshA*	Glycosyl transferase	Val-171-Gly, Aal-187-Val	
Fluoroquinolones	Inhibition of DNA gyrase	DNA gyrase	*M. tuberculosis (2 in 7H10)*	*gyrA*	DNA gyrase subunit A	Ala-90-Val, Asp-94-Gly/Tyr	OFFICIAL, 1990; Cheng et al., 2004; Brown-Elliott et al., 2012; Kasperbauer and De Groote, 2015; Philley and Griffith, 2015
			*M. kansasii (2)*	*gyrB*	DNA gyrase subunit B	Asn-533-Thr	
						RGM (4)	
P-aminosalicylicacid (PAS)	Inhibition of folate synthesis	Thymidylate synthase, Dihydrofolate synthase, Dihydrofolate reductases	*M. tuberculosis (2 in 7H10)*	*thyA*	Thymidylate synthase	Thr-202-Ala, Val-261-Gly	Mathys et al., 2009; Da Silva and Palomino, 2011; Zheng et al., 2013; Zhao et al., 2014; Zhang X. et al., 2015
				*folC*	Dihydrofolate synthase	Glu-153-Aal, Asn-73-Ser	
				*ribD*	Dihydrofolate reductases	G-11-A SNP	
Cycloserine	Inhibition of peptidoglycan synthesis	Alanine racemase, D-Alanine-D-alanine ligase, D-serine/L- and D-alanine/glycine/D-cycloserine proton symporter, L-alanine dehydrogenase	*M. tuberculosis (5–10 in BACTEC)*	*alr*	Alanine racemase	G-10-T SNP	Cáceres et al., 1997; Pelayo et al., 2009; Da Silva and Palomino, 2011; Chen et al., 2012; Gu et al., 2016
				*ddl*	D-Alanine-D-alanine ligase	–	
				*cycA Ald*	D-serine/L- and D-alanine/glycine/D-cycloserine proton symporter	Gly-122-Ser	
					L-alanine dehydrogenase		
Macrolide- ketolide	Inhibition of protein synthesis	50S ribosomal subunit	*M. avium (8–16 in BACTEC)*	*rrl*	23S ribosomal RNA	A-2058-T, A-2059-C SNPs	Meier et al., 1994; Da Silva and Palomino, 2011; Philley and Griffith, 2015
Clofazimine	Interfering with redox	NADH dehydrogenase	*M. tuberculosis (1)*	*rv0678*	Transcriptional regulator	G193 deletion, C-466-T SNPs	Hartkoorn et al., 2014; Zhang S. et al., 2015
	Cycling, causing membrane destabilization and production of reactive oxygen species			*rv2535c rv1979c*	Peptidase Permease	G-265-T SNP T-1052-C SNP	
Oxazolidinones (Linezolid, Sutezolid [PNU-100480] and AZD5847	Inhibition of protein synthesis	50S ribosomal subunit	*M. tuberculosis (4–8)*	*rrl rplC*	23S ribosomal rRNA 50S ribosomal protein L3	G-2061-T, G-2576-T, G-2270-T T-460-C	Hillemann et al., 2008; Williams et al., 2009; Balasubramanian et al., 2014
B-Lactams (In combination with belactamase inhibitor)	Inhibition of peptidoglycan synthesis	Transpeptidases	*M. tuberculosis (NR) RGM (128)*	*blaC ponA Pbp*	beta-lactamase Penicillin-binding Proteins Penicillin-binding proteins	– – –	Fattorini et al., 1992; Hugonnet and Blanchard, 2007; Brown-Elliott et al., 2012; Wivagg et al., 2014
Tetracyclines and glycylcyclines	Inhibition of protein synthesis	30S ribosomal subunit	*RGM (8–16)*	*16S rRNA gene*	16S rRNA	–	Brown-Elliott et al., 2012; Kasperbauer and De Groote, 2015
Trimethoprim and sulfonamides	Inhibition of folate synthesis	Dihydrofolate reductase, Dihydropteroate synthetase	*RGM (64)*	*dfrA sulI and folP1*	Dihydrofolate reductase Dihydropteroate synthetase	–	Brown-Elliott et al., 2012; Kasperbauer and De Groote, 2015
Bedaquiline (TMC207)	Inhibition of ATP synthesis	ATP synthase	*M. tuberculosis (0.25)*	*atpE*	ATP synthase	Ala-63-Pro, Ile-66-Met	Andries et al., 2005; Segala et al., 2012
Nitroimidazoles (PA-824 [Pretomanid] and OPC-67683 [Delamanid])	Inhibition of cell wall lipid and protein synthesis	Dehydrogenase, Nitroreductase	*M. tuberculosis (0.5)*	*Rv0407 Rv3547*	Dehydrogenase Nitroreductase	– –	Stover et al., 2000; Matsumoto et al., 2006; Rivers and Mancera, 2008
SQ109	Inhibition of lipid synthesis	Mycolic acids	*M. tuberculosis (0.5)*	*mmpL3*	Membrane transporter	–	Protopopova et al., 2005; Tahlan et al., 2012
Phenothiazines (Thioridazine and Chlorpromazine)	Inhibition of calcium transport, inhibition of type II NADH	–	*M. tuberculosis (1–32)*	–	–	–	Ordway et al., 2003; Martins et al., 2008
Benzothiazinones (BTZ043)	Inhibition of cell wall arabinans synthesis	Nitroreductase,	*M. tuberculosis (6)*	*nfnB dprE1*	Nitroreductase	–	Makarov et al., 2009; Manina et al., 2010
		Decaprenylphosphoryl epimerase			Decaprenylphosphoryl epimerase	–	
NAS-21 and NAS-91	Inhibition of mycolic acid synthesis	FAS-II dehydratase	*M. tuberculosis (NR)*	*rv0636*	FAS-II dehydratase	–	Gratraud et al., 2008

*RGM, rapidly growing mycobacteria; SNP, single nucleotide polymorphism; NR, not reported*.

In MTB, resistance-associated point mutations, have been described for all first-line drugs (INH, RIF, PZA and EMB), and for several second-line and newer drugs (fluoroquinolones, macrolides and Bedaquiline) (Somoskovi et al., 2001; Da Silva and Palomino, 2011; Segala et al., 2012).

#### Acquired resistance to first-line TB drugs due to mutations

This is considered as the principal mechanism of resistance to INH and RIF, the most powerful anti-TB agents (see Table 3). Resistance to INH is a complex process. Mutations in several genes, including *katG, inhA, ahpC, kasA*, and *ndh* have all been associated with INH resistance (Da Silva and Palomino, 2011). INH is a pro-drug requiring activation by the catalase/peroxidase enzyme encoded by *katG*. A reduction in catalase/peroxidase activity as a result of *katG* mutations is the most common mechanism associated with INH resistance. Other such mechanisms involve mutations in the *inhA* promoter that result in overexpression of *inhA*, which confers low-level resistance to INH (Cohen et al., 2014). More than 80% of INH resistance cases could be explained by the two mutations of *katG* S315T and *inhA* promoter -15C-T (Torres et al., 2015). Recently, Torres et al, discovered novel mutations that are able to explain 98% of INH resistant phenotypes by a *katG, inhA* promoter, or a *fabG1* mutation (Torres et al., 2015). Another important finding about the mutations associated with INH resistance is that the identification of harbinger mutations, such as *katG* S315T, may serve as an early warning signal for MDR emergence (Manson et al., 2017). The finding has a major public health impact as it can enable targeted treatment of patients with pre-MDR-TB.

For RIF, more than 95% of resistant strains have a mutation within the 81-bp hotspot region (codons 507–533) of the RNA polymerase beta-subunit gene (*rpoB*) (Nasiri et al., 2016).

PZA is the backbone of short-course chemotherapy for TB, which is an important drug to shorten treatment regimens. Mutations in the *pncA* gene result in the loss of pyrazinamidase activity and have strong correlation with PZA resistance (Gu et al., 2016). Studies also indicated that mutations in the *rpsA* (encode the ribosomal protein S1) and *panD* (encodes aspartate decarboxylase) genes are responsible for PZA resistance (Shi et al., 2011, 2014). In addition to above mentioned genes, Zhang et al. showed that mutations in a newly identified gene *clpC1*, which encodes an ATP-dependent ATPase involved in protein degradation is associated with PZA resistance in MTB (Zhang et al., 2017).

EMB, another first-line anti-TB drug, together with INH, RIF, and PZA, currently is used for treatment of TB and prevents the emergence of drug resistance. Several studies have shown that mutations in the *embCAB* operon (encoding arabinosyltransferases in MTB) particularly the *embB* gene, are a major cause of EMB resistance in MTB (Lingaraju et al., 2016). Mutations in the *ubiA* gene also appear to be responsible for high-level EMB resistance in MTB (Safi et al., 2013; He et al., 2015; Lingaraju et al., 2016).

#### Acquired resistance to second-line TB drugs due to mutations

Kanamycin and amikacin are the major aminoglycosides used for treatment of MDR-TB and several other mycobacterial species. These antibiotics inhibit protein synthesis by binding to the 30S subunit of the mycobacterial ribosome. The most common mechanism of resistance to aminoglycosides has been associated with an A1401G mutation in the *rrs* gene coding for 16S rRNA (Da Silva and Palomino, 2011). Unlike most other bacteria, which have multiple copies of the *rrs* gene, mycobacteria have a single copy of the gene (Cohen et al., 2014). Consequently, mutations in this gene are usually associated with high-level aminoglycoside resistance. Capreomycin and viomycin are polypeptide antimicrobial agents that are also used in combination therapy for treatment of drug-resistant MTB strains (Maus et al., 2005a). Like the structurally unrelated aminoglycosides, capreomycin and viomycin are bactericidal drugs that inhibit protein synthesis. MTB strains that acquire resistance to kanamycin, usually become resistant to capreomycin or viomycin (Maus et al., 2005a). However, cross-resistance between kanamycin and capreomycin and between kanamycin and viomycin is variable (Musser, 1995). Mutations in the *rrs* gene have also been implicated in resistance to capreomycin and viomycin (Jnawali et al., 2013).

Resistances to quinolones are also associated mainly with mutations in drug target genes. Fluoroquinolones are second-line anti-TB agents, and they currently form the backbone of MDR-TB therapy. They are also used for treatment of mycobacterial infections caused by *M. kansasii, M. simiae*, and *M. fortuitum* (OFFICIAL, 1990). Quinolones target two essential bacterial type II topoisomerases, DNA gyrase (also known as topoisomerase II) and DNA topoisomerase IV, enzymes that regulate the supercoiling of DNA and are thus essential for bacterial DNA replication and cell division (Drlica, 1999). While many bacterial species contain both DNA gyrase and topoisomerase IV, mycobacteria lack topoisomerase IV and contain only DNA gyrase, a tetramer consisting of two A and two B subunits, encoded by the genes *gyrA* and *gyrB*, respectively (Cole et al., 1998). Different bactericidal activity of various fluoroquinolones may be explained by their specificity for different enzymes; ciprofloxacin, which is less effective against MTB, preferentially targets topoisomerase IV, which is lacking in MTB, whereas newer-generation fluoroquinolones including moxifloxacin and levofloxacin, has a predilection for DNA gyrase (Ginsburg et al., 2003). Fluoroquinolone resistance in mycobacteria is associated with mutations within a highly conserved region, the quinolone resistance-determining region (QRDR), of the *gyrA* and *gyrB* genes (Takiff et al., 1994; Ginsburg et al., 2003). The most common mutation in fluoroquinolone-resistant MTB isolates involves a substitution at codon 90 and 94 of the *gyrA* gene (Von Groll et al., 2009; Sirgel et al., 2012). Specific amino acid changes in the QRDR can cause distinct levels of quinolone resistance. A high-level resistance is often associated with at least two mutations in *gyrA* or mutations in *gyrA* plus *gyrB*, with double *gyrA* mutants expressing the highest level of resistance (MIC 20 mg/mL) to sparfloxacin (Ginsburg et al., 2003). As indicated by previous studies, fluoroquinolone resistance is also mediated by efflux mechanism (Pasca et al., 2004; da Silva et al., 2011; Li et al., 2014).

Macrolides are bacteriostatic antibiotics that inhibit protein synthesis in a wide range of bacteria by binding to the 50S ribosomal subunit. Species of mycobacteria belonging to MTB complex (MTC) are intrinsically resistant to macrolides and different members of this class of antibiotics have little or no effect on MTC.

Linezolid, the main oxazolidinone currently in clinical use, is most commonly used to treat drug resistant TB, but its use has been limited by toxicity concerns. Mutations in *rplC* gene encoding for 50S ribosomal protein L3 and *rrl* gene encoding for 23S rRNA have been detected in linezolid-resistant clinical isolates (Hillemann et al., 2008; Beckert et al., 2012).

Resistance to new anti-TB drugs has also been documented. Bedaquiline (Sirturo, TMC207), is a diarylquinoline drug that was recently approved for use against MDR-TB. It binds to and inhibits the mycobacterial ATP synthase encoded by the essential gene *atpE* (Andries et al., 2005). Resistance to Bedaquiline is mediated by mutations in the *atpE* gene, typically at positions 63 or 66 (Petrella et al., 2006). However, resistant mutants have been identified that lack any mutation in the *atpE* or in the other genes encoding components of ATP synthase, indicating alternative mechanisms of drug resistance (Huitric et al., 2010). Recently, mutations in transcriptional repressor (Rv0678) in Bedaquiline resistant MTB isolates without *atpE* mutations have been recognized (Andries et al., 2014; Hartkoorn et al., 2014; Zhang S. et al., 2015). These mutations resulted in the upregulation of mmpL5-mmpS5 expression, thereby leading to increased efflux and cross-resistance to Clofazimine (Andries et al., 2014; Hartkoorn et al., 2014; Zhang S. et al., 2015).

#### Mutations conferring resistance to drugs used to treat NTM

Unlike TB, disease caused by NTM, is rarely treated by first-line anti-TB drugs. For the treatment of NTM infections, the macrolide antibiotics play an important role in the therapeutic regimens (van Ingen et al., 2012). Since the strains from the MTC are intrinsically resistant to macrolides, the problem of emergence of macrolide resistance will be discussed only for NTM (Doucet-Populaire et al., 2002; Buriánková et al., 2004). Single point mutation in position 2058 or 2059 of the 23S rRNA gene (*rrl*) has been associated with high level macrolide resistance in several clinical isolates of NTM, including *M. abscessus, M. avium* complex (MAC), *M. chelonae, M. fortuitum*, and *M. kansasii* (Meier et al., 1996; Wallace et al., 1996; Burman et al., 1998; Bastian et al., 2011; Brown-Elliott et al., 2012).

Resistance to macrolides in MAC poses a significant challenge to effective prophylaxis and treatment outcome in HIV infected patients (Griffith et al., 2007). In these patients, macrolides are used for MAC infection prophylaxis. Macrolide-resistant isolates of MAC have been found in most patients with unsuccessful macrolide prophylaxis. Emergence of resistance to macrolides during treatment has been also described for other NTM species (Doucet-Populaire et al., 2002). According to the current American Thoracic Society guidelines, macrolide antibiotics (clarithromycin and azithromycin) must be administrated in combination with other drugs to prevent the emergence of macrolide resistance in NTM species (Griffith et al., 2007).

The primary mechanism of acquired resistance to aminoglycosides in NTM is based on mutations in the 16S rRNA gene. A mutation in position 1408 of the 16S ribosomal RNA (*rrs*) gene is responsible for high-level resistance in both *M. abscessus* and *M. chelonae* after therapy as well as *in vitro* selection (Wallace et al., 1985; Prammananan et al., 1998). *Mycobacterium abscessus* represents one of the most common antibiotic-resistant RGM species, which is usually resistant to major anti-TB drugs, as well as most antimycobacterial drugs, including tetracycline, fluoroquinolones and sulphonamides (Nessar et al., 2011). However, this species is naturally susceptible to amikacin and clarithromycin/azithromycin, which are used in combination for treatment of infections caused by this bacterium (Petrini, 2006). Thus, emergence of amikacin-resistant isolates of *M. abscessus* could complicate the management of these infections.

Fluoroquinolones appear to be drugs with therapeutic possibilities against clinical isolates of *M. fortuitum, M. chelonae*, and *M. kansasii* (Dıaz et al., 2003). In NTM species, the most common acquired resistance mechanism to fluoroquinolones involves a stepwise accumulation of mutations in the QRDR of *gyrA* gene (Brown-Elliott et al., 2012; Monego et al., 2012).

RIF is the key component of treatment regimens for diseases causes by *M. kansasii* and MAC (Klein et al., 2001; Obata et al., 2006; Brown-Elliott et al., 2012). Acquired RIF resistance in *M. kansasii* and MAC has been documented and is conferred primarily by mutations in the *rpoB* gene (Klein et al., 2001; Obata et al., 2006). These mutations are identical to those observed in RIF-resistant MTB isolates (Brown-Elliott et al., 2012).

## Conclusions

The infections due to *Mycobacterium* species, particularly strains that are clinically important and resistant to clinical agents, poses significant public health problems. Reports about drug resistant mycobacteria from different countries, suggest that common mycobacterial species may become refractory to any chemotherapeutic agent in the future (Velayati et al., 2009; Migliori et al., 2012; Udwadia et al., 2012; Klopper et al., 2013).

The limited number of new anti- mycobacterial agents coming to market and new threats arising from drug resistant isolates (including MDR and XDR isolates) brings us to the end of the “antibiotics era.” *Mycobacterium* species including MTB are armed with a wide variety of intrinsic and acquired drug resistance mechanisms: Modification of antibiotic targets mediated by bacterial specific enzymes or mutations and degradation/modification of antibiotics by production of antibiotic inactivating enzymes renders the *Mycobacterium* species resistant to most classes of antimicrobials. Several mycobacterial drug efflux pumps have been identified in INH and RIF resistant isolates providing resistance by expelling the drug molecules that enter the cell. Besides, unique structural and physiological properties of MTB including low cell envelope permeability, slow growth rate, and the ability to enter a metabolically inactive dormant phase have even exacerbated the drug resistance problem leaving us perilously close to none or very limited number of therapeutic options. This unavoidable circumstance emphasizes the urgent need for the development of multidisciplinary approaches to combat the resistance crisis. Modification of existing antibiotics and screening for new antibiotics from unexplored ecological niches that can act on novel targets and inhibit existing resistance mechanisms such as inhibitors of efflux pumps, factors involved in dormancy induction and bacterial enzymes involved in cell envelope synthesis, target modification or antibiotic inactivation/modification provide promising strategies to disarm the resistant *Mycobacterium* species. To this end, understanding the mechanisms involved in mycobacterial tolerance to antibiotics could aid not only for planning an appropriate therapeutic regimen but also for development of novel therapeutic agents that can overwhelm existing resistance mechanisms. In recent years, due to the advances in genomics and biology, our knowledge of the remarkable diversity of mechanisms of antimicrobial resistance in *Mycobacteria* has greatly increased. However, despite substantial progress, there is clearly much work to be done to fully elucidate the molecular basis of antimicrobial resistance in this group of bacteria. The best hope for the future is the development of a greater understanding of exact mechanisms of antimicrobial resistance in mycobacteria to further improve the therapeutic outcomes in *Mycobacterium* infected patients.

## Author contributions

Conceived and designed the study and wrote the paper: MJN. Participated in manuscript revising and editing: MJN, MH, MG, HG, AP, AAIF, and MMF.

### Conflict of interest statement

The authors declare that the research was conducted in the absence of any commercial or financial relationships that could be construed as a potential conflict of interest.
